# President’s Address before the Chicago Gynæcological Society at the Second Annual Meeting

**Published:** 1881-01

**Authors:** W. H. Byford


ARTICLE II.

PRESIDENT'S ADDRESS. By W. H. BYFORD, M.D., before the Chicago Gynæcological Society, at the Second Annual Meeting, Nov. 15, 1880.

GENTLEMEN FELLOWS: By your partiality in electing me President of the Chicago Gynæcological Society, you have placed me under an obligation for an address.

In considering the matter of a subject it has seemed to me proper to devote the short time I propose to occupy in tracing, in a concise way, the history of gynæcology in this city. I do not design to do more than give a sketch or outline of the subject, leaving those present to fill up the deficiencies.

If I should say less about obstetrics and diseases of children than of the diseases of women, I am sure I shall be excused, for the double reason, that I know less about them, and that gentlemen more interested in those departments of study will some day be called upon to fill up the hiatus by having the office of president thrust upon them.

It seems hardly creditable that gynæcology as a special study has been almost wholly developed within my recollection. When I studied medicine, the diseases of women outside of those immediately connected with pregnancy and child-bed were very imperfectly understood; and in the lectures by teachers in the colleges did not occupy half a dozen days. And while general practitioners knew very little of them, because they were not taught, and because there were no books upon the subject, they were very little behind the professors. It was not till a short time before I came to Chicago that gynæcology began to assume definite shape. Up to 1845, the French, through three or four illustrious teachers, as Lisfrane, Recamier and Vulpean had arrived at unusual correctness in diagnosis, and consequently, for those times, great efficiency in treatment.

In 1845, Dr. J. H. Bennett, who had been interne in the French hospitals, and had become thoroughly imbued with the teachings of the Great Masters in the French capital, published the first edition of his works on the uterus, and by his positiveness, perspicuity of style and enthusiasm captivated the young men in the profession. It was so comforting to the junior practitioner to be led by demonstrative facts out of the maze of uncertainty into a field of tangible reality, that many embraced his doctrines and confidently committed themselves to his practice. An impetus of no mean importance was given in this country to the progress of the ideas promulgated through Bennett by Prof. C. D. Meigs, himself an enthusiast, embracing and urging a similar course of teaching.

Shortly previous to, at the time of, and subsequent to the publishing of Bennett's books, Simon and Koeberle, respectively of Germany and Alsace, were exhibiting equal fervor in the cultivation of our branch of medical science, and Simpson, of Scotland, was grafting upon the old English stem so many novelties as to yield an entirely different fruit to that it had ever borne before; and our own immortal Sims and Atlee were more quietly yet not less efficiently working surgical problems that overturned the whole gynæcological fabric of former times and established upon its ruins not dogmatic but rational gynæcology.

The harvest of such vast efforts afforded work for many reapers; and there arose in Germany such men as Carl Braun, the elder and junior, Martin Schroeder, Scanzoni and a host of others, Tyler Smith, Barnes, Hewitt, Atthil, Spencer Wells and many more in England; while Peasley, Thomas, Emmet, Goodell, Battey and others entered the list in this country. Then the race began and never at any time have such strides of progress been made by any body of men as the gynæcologist of the last quarter of a century. The field of research was new, inviting and remunerative; the men were young, energetic learners, and ambitious, and if at first they started off as disciples of some particular school, they soon became masters; their labor assumed such an individuality and often originality that created an accumulating store of knowledge which will mark this age in gynæcology with as durable, glorious and much more beneficent fame than the literature of the age of Elizabeth characterises that period of English history.

The gynæcologists of our time have availed themselves of all that is valuable in the general subject of therapeutics and surgery, and the special subjects springing from them. The microscope, stethoscope, thermometer, sphygmograph, test tube, in fact all the instruments used in the rudimentary and practical branches of medicine, besides those peculiar to their own branch of practice, have been turned to advantage. I dare not try to give you all the particular improvements which have grown out of and been appropriated by those engaged in this hot pursuit of knowledge. Ovariotomy, oöphorectomy, hysterotomy, hystereotomy, tracheloraphy, perineoraphy, the cure of vesico-vaginal fistula and many others of the most difficult and most beneficent operations, most of them originating with the gynæcologist, and the remainder plucked from the category of general surgery, bound in our magnificent fasciculus, and given the title of gynæcological surgery.

Perhaps, gentlemen and fellows, it may be necessary for me to apologize for this digressive panegyric of our subject, but as so much of these things have transpired during my time and as in them I have been an humble but interested participator, I venture to hope you will not require it of me.

Now, to come down to the gynæcology of Chicago. Before entering into the details allow me to say something of the different schools of gynæcology, imperfect as they were when I commenced my labors in this city. There were at least three different and well pronounced theories of uterine disease, which you know, after all, form the main bulk of our practice.

The French theory was, that inflammation and ulceration were the prime conditions of all uterine cases, and all the accompanying states of the organ the consequences, and were to be cured by local treatment. The teachings of this school, as I have shown you, was first given to the English speaking peoples by

Dr. Bennett, and had gained great ascendancy at that time in this country. The English school, represented by Bigby and the earlier works of West, promulgated the doctrine that the diseases of the uterus were the consequence of general disorder of the system, and that general treatment of an appropriate character would cure them.

The American school, as represented by Chapman, Dewees and Hodge, the renowned trio of the University of Pennsylvania, taught that displacements of the uterus were the prime and governing factors in uterine ailments, and that support was the great requisite. I need not tell you that the teachings of this school is perpetuated, and one of its glorious old masters immortalized, by the invention of Hodge's pessary. In some of its many forms it has everywhere prevailed and carried with it the name of Hodge into every civilized community on the globe. It has imprinted its likeness upon almost every useful instrument now employed; and I cannot now see why it will not forever be indispensable to the intelligent exercise of the art of gynæcology.

None of these exist as a separate school in this country at the present time, but all are judiciously blended in one system to the great benefit of the suffering women of this period.

Some other theories of female disease, not so generally accepted, were taught by men of eminence. Drs. Tyler Smith and Tilt looked upon diseases of the ovaries as the source of many other diseases and disorders of the genital system; and according to these learned gentlemen the sympathetic influence of the female genital system was reflected from these organs in a state of disease. There is great plausibility in this theory, inasmuch as the ovaries are now acknowledged as the presiding, regulating and actuating organs of that system.

In 1857, when I came here, Dr. John Evans had just resigned the position of Professor of Obstetrics and Diseases of Women and Children in Rush Medical College, and was the only professional gentleman in the city, so far as I know, who either had or deserved to have any reputation as a gynæcologist. He had taught the branch to which he had been elected, very acceptably, and would no doubt have made a wider and more enduring fame if he had not turned his attention to politics and building railroads. The professional reputation he left behind him, however, is one of which he may justly be proud. At that time gynæcology was cultivated by all the intelligent members of the profession in this city to the limited extent necessitated by the want of facilities for studying it. As compared to its present condition, the subject did not deserve and had not attained to the dignity of a separate name. The appliances used in the treatment of diseases of women were very imperfect, and the gravity with which they were employed would have been amusing if they had not often been mischievous. The cylinder was the representative speculum, although the bivalve and quadrivalve were occasionally seen as curiosities. I have several times seen a lamp chimney do service as a cylindrical speculum, and heard the owners congratulate themselves upon the simplicity of these instruments as compared to the fancy and expensive articles of which they had heard. As the practitioners of this city generally accepted the teachings of Bennett, a caustic holder was a necessary part of their equipment, and goose-quills served their purpose admirably. I assure you in all earnestness that I have known men to practice gynæcology to their own satisfaction with those two instruments alone. It is not in a spirit of derision that I tell you these facts, connected with the gynæcology of those times, but for the purpose of giving you an idea of its rude condition.

Two years after the resignation of Dr. Evans, our accomplished fellow, Dr. DeLaskie Miller, was elected to fill the same chair in Rush Medical College, and I have often wondered at and admired his courage and perseverance in discharging the duties of three men in that institution for so many years. That those duties were conscientiously performed, no one will doubt; that they were acceptably rendered, the fact that he has lectured to more students on that subject than any other man in the Northwest, with possibly one exception, sufficiently attests. To him belongs the credit of establishing the first gynæcological clinique in this city. This clinique was successfully conducted by him at Rush Medical College on the North Side, as far back as 1862. All who knew him will join me in wishing a long continuance of his useful career.

In 1859 Chicago Medical College was organized, and an incumbent to the tripod chair of Obstetrics and Diseases of Women and Children elected. The duties of this laborious position were divided with Dr. E. O. F. Roler in the year 1868. Twelve years seem but a very short time, and there is so much of an advancement since then that we forget the fact that, if not the first, it was among the first instances in which this step was taken.

Dr. Roler has filled the chair of Obstetrics in a manner that has placed him in the foremost ranks of obstetricians in the Northwest, if not in America.

In 1872 an organization called the Chicago Obstetrical Hospital was effected. This organization occupied a part of the Chicago Medical College building, but was otherwise independent of it. The members of this institution were Dr. Daniel T. Nelson, who did the first work that led to its foundation, Drs. E. O. F. Roler, H. T. Merriman, H. T. Byford and myself. The clinical gynæcological teachings of the Chicago Medical College were conducted by the members of the Chicago Obstetrical Hospital up to last year. The property of that institution is now in the keeping of the Woman's Hospital of the State of Illinois. I am happy to recognize in the gentlemen connected with that institution, gynæcologists who are a credit to our city and society and whose future is full of promise.

In the year 1870 the Woman's Hospital Medical College was organized, and the separate chairs of Obstetrics, Gynæcology and Diseases of Children were established as a part of its original organization. Dr. Eugene Marguerat was elected to the chair of Obstetrics. For several years he discharged the duties connected with his position in a painstaking, faithful and very acceptable manner, and is now recognized as an able practitioner of obstetrics.

Our sturdy and indefatigable fellow, Dr. T. Davis Fitch, became Professor of Gynæcology, and still occupies the position in that institution. It is not in a spirit of flattery that I say in his presence, Dr. Fitch has already achieved a national reputation as a gynæcologist, and that the Woman's Medical College owes much of its present highly prosperous condition to his devotion, industry and ability.

The chair of Diseases of Children was given to Dr. Chas. G. Smith. I need not speak to you of Dr. Smith's well known acquirements in this direction—they are too familiar to you to require it. Dr. Smith's large and increasing practice was too exacting to permit him to retain that chair. He was succeeded by Dr. Mary H. Thompson, who also very soon retired from the place, to more completely devote her whole time to the Hospital for Women and Children. Our fellow, Dr. Chas. W. Earle, was transferred to this chair, and has ardently, intelligently and devotedly discharged the duties of it to the present time. His usefulness as a member of the faculty is second to no other belonging to the college, and he has only to persevere in the way he now goes to occupy a high position in this great city and country.

I have yet to chronicle another event in the history of our colleges in which some of our fellows have played a conspicuous part. I refer to the organization of a Spring Faculty in the Rush Medical College. This occurred in the year 1872, and the choice of members of the faculty was determined by *concours*. In that *concours* the contest was participated in by some of the ablest men in this city and State. Our fellow, Dr. A. Reeves Jackson, triumphantly won the chair of Gynæcology, and Dr. E. Warren Sawyer that of Obstetrics. It is only the truth to say that the brilliant success of this enterprise was largely due to the able and acceptable teachings of these two talented gentlemen.

About 1862 the Hospital for Women and Children was established and Dr. Mary H. Thompson was appointed by the board of trustees "Head Surgeon." She still occupies that position, having held it continuously from that time. To this position she owes much of her uncommonly successful and useful career. Dr. Thompson has performed almost all the difficult and important operations in gynæcological surgery, and deservedly ranks among the most successful gynæcological practitioners. Dr. Sarah Hackett Stevenson is associated with Dr. Thompson in this hospital, and a bright future awaits her.

About the same period, for the first time, the Mercy Hospital was open to gynæcological cases. Later, cases of obstetrics were received into the same institution. Since then the department of obstetrics and gynæcology has developed into a useful field for teaching, and has been fully utilized by the Chicago Medical College.

In August, 1871, a charter was issued to Dr. A. Reeves Jackson and associates, for the organization of the Woman's Hospital of the State of Illinois. It is well known that this enterprise originated with Dr. Jackson, and through his untiring exertion was pushed forward to success. The hospital is still in existence, and it is hoped will become one of the enduring institutions of Chicago—the means of great good to suffering women, and a source from which will emanate much useful gynæcological knowledge.

When Cook County Hospital was organized in the year 1855, there was not a distinct gynæcological department established, and patients laboring under diseases peculiar to women, and even obstetrical cases, were at first placed in charge of one or more physicians of the hospital. Soon, however—probably in 1865—the authorities of the hospital designated certain wards for obstetrical cases and for diseases peculiar to women, and gave Dr. H. W. Jones charge of the department thus created. To Dr. Jones is due the credit of being the first to give regular special *hospital* clinics in gynæcology in Chicago. Later in the history of this hospital, Dr. T. D. Fitch was associated with Dr. Jones in charge of this department.

Our fellows, Drs. H. Webster Jones and T. Davis Fitch, had charge of the department of the hospital for several years—Dr. Fitch until within about two years. Dr. Jones, on account of a large and growing gynæcological and obstetrical practice, resigned his position on the staff of the hospital. These positions are now occupied by gentlemen of great respectability, Drs. Steele and Guerin, and the department under their management is utilized to much advantage for teaching. Our fellow, Dr. Jones, may be regarded as a pioneer gynæcologist in Chicago, having been here twenty-two years, and growing with the city and profession, now enjoys a position of which we as fellows and associates are proud.

In the interval between the year 1857 and now, but especially since the fire, several important dispensaries have been organized, some for gynæcological patients alone and others in which this department of our profession has been well represented. I have already spoken of the one connected with the Mercy Hospital and Chicago Medical College. One for the exclusive treatment of diseases of women and children was and is now connected with the Hospital for Women and Children. Under the vicissitudes caused by poverty, the fire and the panic, this gynæcological dispensary has been kept up, and while dispensing good to the people, has been utilized to great advantage for the purpose of teaching. Connected with the Woman's Hospital of the State of Illinois, there has also been maintained a dispensary for the treatment of diseases of women. Three years ago the Women's Christian Association established a dispensary for the treatment of the diseases of women and children. It has been judiciously managed, and is the source of much good to the suffering poor. All the mixed dispensaries have gynæcological departments, as the German, North Star, and the Central Free Dispensary. This last named dispensary, known for some years as the Brainard Free Dispensary, deserves more particular mention than any of the others named. It is now located on the corner of Wood and West Harrison, in the Rush Medical College building. The gynæcological department of this dispensary has been conducted in an efficient manner by Drs. Adolphus and our fellow Etheridge. It is now in charge of Drs. Adolphus, Fitch and Nelson, is attended by a large number of patients, and is affording material for a very useful clinique. In connection with this dispensary and its gynæcological department, Dr. Adolphus deserves to be honorably mentioned. It is acknowledged by all who are acquainted with the work done there, that the great success of that department, and indeed of the whole dispensary, is due more to the devotion and ability of him than any other one individual.

Gynæcology in Chicago has lately been the recipient of two valuable accessories. I allude to the arrival among us of our accomplished fellows Dr. Dudley and Professor Jenks. They are both so well known to all of you that I need say but little of them. They have both impressed themselves upon the literature of the profession in other cities than this, and have won an honorable position among us. We welcome them with fraternal greetings.

In this very cursory and imperfect sketch of gynæcology in Chicago as I remember it, no doubt I have omitted institutions and individuals that ought to have been mentioned. If any such omission may appear, I beg the indulgence of any that may seem to be slighted, and assure them that at no distant time some one else will do the task in a more complete and graceful manner. I venture to hope, however, that this outline contains the main landmarks as starting points for a connected and full summary of the whole subject.

Chicago twenty-five years ago was a city of less than fifty thousand inhabitants; its houses were nearly all of them made of wood mounted on stilts, had been moved or were in a moving condition; its streets were formed of that native compound called Illinois mud, and in some places it was a long distance to the bottom. The sidewalks resembled irregular stairways, as we could not walk upon them without ascending and descending steps. Now it is a city of half a million inhabitants. Its streets are formed by magnificent structures surpassed by no city in the world—splendid public buildings, such as we visit, when abroad, as curiosities. Its business enterprise and vast resources are wonders to all civilized nations. How will the growth of gynæcology compare with this grand record? Does gynæcology keep pace with the magnificent progress made by commerce in this great Western metropolis? I dare not approach the comparison, but I think all will admit that its growth, taken altogether, is such as to inspire us with pride and courage. Pride that outside of the three great cities of New York, Philadelphia and Boston, we lead the profession. Indeed, I do not know that we ought to place the two last named cities before ours in gynæcological prestige. We should take courage at what we have done, at our increased strength, but above all at our own harmony and organization. Can we not hope that with the formation of this Society an era has been reached from which our progress will be more rapid than ever before?

